# Impact of COVID-19 on the Mental Health of Elderly Patients at Midnapore Medical College, West Bengal

**DOI:** 10.7759/cureus.65169

**Published:** 2024-07-23

**Authors:** Syed Roshan Ali, Manas Patra

**Affiliations:** 1 Community Medicine, Midnapore Medical College, Midnapore, IND

**Keywords:** bangla version, rural, india, geriatric, coping strategies, elderly, stress, anxiety, depression, depression anxiety and stress scale (dass-21)

## Abstract

Background

Depression, anxiety, and stress are leading causes of disability worldwide and major contributors to suicide. The burden of these disorders among the Indian geriatric population is often described as a silent epidemic. The sudden emergence of the COVID-19 pandemic has only intensified this public health problem. Finding out factors associated with poor mental health is critical to improving overall healthcare for high-risk patients, especially in underserved and inaccessible communities.

Aim

This study was conducted to measure the prevalence rates of depression, anxiety, and stress and their sociodemographic correlates among the Indian geriatric patient population. This study also aimed to assess the coping strategies employed and difficulties faced by the population during the COVID-19 pandemic.

Methods

A cross-sectional survey was conducted using a pre-designed and pre-tested questionnaire. A total of 107 participants were recruited through convenience sampling. Depression, anxiety, and stress were measured using the Bangla version of the Depression, Anxiety, and Stress Scale (DASS-21 BV), a 21-item self-reported questionnaire.

Results

Of the sampled group, 43.9%, 32.7%, and 34.6% were moderately to extremely severely depressed, anxious, and stressed, respectively. Factors associated with worse mental health were increasing age, female gender, living separately from their spouses, unemployment, retirement, or any occupation that did not require one to leave their house. Of the sample population, 80.3% had experienced a loss of income due to the pandemic. The most frequently used coping strategy was to solve problems they faced daily, closely followed by praying and participating in religious activities.

Conclusion

Depression, anxiety, and stress showed a higher prevalence than previously described, before the pandemic. This could be due to the effects of the COVID-19 pandemic. Our study also demonstrated some of the factors associated with and the most commonly used ways to tackle poor mental health. Adequate educational awareness programmes that are accessible in different regional languages, strengthening mental health infrastructure, and community mental health services will significantly improve outcomes, especially among high-risk populations.

## Introduction

Mental disorders, including depression and anxiety, contribute significantly to the global burden of disease [1]. In fact, depression is the leading cause of disability worldwide and a major contributor to suicides, with an alarming annual rate of approximately 800,000 cases [2]. With declining fertility rates and increasing life expectancy, the elderly population of India (i.e., aged 60 years or more) is steadily increasing. As per the prevailing socially sanctioned roles for this segment of the population, they are more susceptible to these mental disorders and yet remain undiagnosed. Often, symptoms of mental illness are disregarded both by the patient and their family as part of the “normal ageing process” or something “not so serious” [3]. Consequently, the burden of mental disorders among the geriatric population in India has been characterised as a silent epidemic [3]. 

The sudden emergence of the COVID-19 pandemic against this backdrop has created an environment in which many determinants of mental health are also affected. These include separation from loved ones, loss of livelihood, social isolation, fear of infection, inadequate basic supplies (eg. food, water, clothes, or accommodation), and social stigma associated with those infected by the virus. The mortality rate and severity of infection among older adults further increase their vulnerability to mental illness [4]. 

Despite these critical implications, there is a dearth of information regarding the impact of the pandemic on geriatric mental health in India. Moreover, individuals who exclusively speak regional languages are often excluded from mental health studies, leading to potential misdiagnoses and inappropriate treatment [5]. In this context, the present study aimed to investigate the prevalence of depression, anxiety, and stress among the elderly population using a Bangla questionnaire, specifically tailored to the local population of West Bengal, India.

The objectives of the study were to know the prevalence of anxiety, depression and stress among the elderly, find out the different sociodemographic and pandemic-related factors associated with these mental health problems, and elicit the coping strategies used by the participants to deal with these problems.

## Materials and methods

This was a cross-sectional descriptive epidemiological study of elderly patients, aged 60 years or more, attending the outpatient departments of different disciplines in Midnapore Medical College and Hospital (MMCH), Paschim Midnapore, West Bengal, India. The study was conducted at MMCH from August 2023 to February 2024. The study was approved by the Government of West Bengal Institutional Ethics Committee Midnapore Medical College (approval number: IEC/2023/5/(9) dated August 17, 2023). All data was collected and analyzed, maintaining strict confidentiality, after obtaining informed consent from the study participants.

Sample

Participants were recruited using non-random purposive sampling. Patients who gave written informed consent, were not attending the psychiatry OPD, and were not seriously ill were included in the study. Considering the prevalence of depression among the elderly in India as suggested by Pilania et al. [2] as 34.4%, a total of 107 people were interviewed. An absolute error of 10% and a standard normal variate (Z) taken as 1.96 (at 95% confidence interval (CI)) were used. Data was collected for a period of 10 days spread over two weeks, with approximately 10 participants being interviewed daily, by the principal investigator.

Data collection

Data was collected through a semi-structured interview using a pre-designed and pre-tested questionnaire with three sections (See Appendices). The first section included the various relevant sociodemographic variables, namely age, sex, marital status, education, type of family, per capita income, religion, caste, occupation, area of residence, and comorbidities present. The second section elicited the occurrence of some common problems faced during the pandemic including history of COVID-19 infection, hospitalization of self, hospitalization of a loved one, loss of a loved one, loss of income, experience of social isolation/shunning, fear of a COVID-19 outbreak, and how the participants tackled these problems. The first two sections were finalized after consultation with experts and were pre-tested on a small number of participants attending the various OPDs at MMCH. The third section was the Bangla version of the Depression, Anxiety, and Stress Scale (DASS-21 BV) consisting of 21 items, previously tested and validated by Alim et al. [6]. It was used after obtaining prior permission from the author. The scale is divided into three sub-scales with each containing seven items. Participants were asked to score every item on a scale from 0 (“did not apply to me at all”) to 3 (“applied to me very much”). Cumulative scores were calculated for each sub-scale by adding up the respective scores and multiplying them by a factor of two. Cut-off scores for conventional severity labels (i.e, normal, mild, moderate, severe, and extremely severe) as suggested by The DASS-Manual [7] and a number of subsequent studies [8,9] were utilised. As found by Wood et al., 2010 [10], the DASS-21 is a suitable and reliable instrument for measuring depression, anxiety, and stress in patients over 60 years of age. The DASS-21 questionnaire also has the benefit of an extremely high completion rate, with the 21-question format minimising participant fatigue, thereby being especially useful for the oldest participants among the elderly population [10]. 

Statistical analysis

The collected data was coded, entered, and analysed by using Microsoft Excel (Microsoft Corporation, Redmond, Washington, United States), IBM SPSS Statistics for Windows, Version 23.0 (Released 2015; IBM Corp., Armonk, New York, United States), and Python (Python Software Foundation, Wilmington, Delaware, United States). It was normally distributed. The association between independent and dependent variables was determined by using Pearson’s chi-square test. A p-value <0.05 was considered significant. 

## Results

A total of 107 participants were interviewed. There were 57 males (Mean age = 66.42, SD = 7.08, range = 60-92 years) and 50 females (Mean age = 68.20, SD = 7.97, range = 60-89 years). The sample characteristics are given in Table 1.

**Table 1:** Baseline sample characteristics ^According to the Revised Modified BG Prasad Socioeconomic Status Classification for October 2023 [11].

Variables		Frequency (Percentage)
Sex	Male	57 (53.27%)
	Female	50 (46.73%)
Age (years)	60-70	78 (72.90%)
	70-80	20 (18.69%)
	80+	9 (8.41%)
Education Status	Never went to School	31 (28.97%)
	Primary School	48 (44.86%)
	High School	21 (19.63%)
	College	7 (6.54%)
Religion	Hindu	67 (62.62%)
	Muslim	40 (37.38%)
Area of Residence	Urban	29 (27.10%)
	Rural	78 (72.90%)
Socio-Economic Class^	Upper Class	6 (5.61%)
	Upper Middle Class	8 (7.48%)
	Middle Class	7 (6.54%)
	Lower Middle Class	26 (24.30%)
	Lower Class	60 (56.07%)
Status of Spouse	Living together	73 (68.22%)
	Living separately/ Dead	34 (31.78%)
Type of Family	Joint	53 (49.53%)
	Nuclear	54 (50.47%)
COVID-19 consequences	Symptomatology	12 (11.21%)
	Hospitalization	10 (9.35%)
	Hospitalization of a loved one	19 (17.76%)
	Loss of a loved one	13 (12.15%)
	Loss of Income	86 (80.37%)
	Experienced social shunning	21 (19.63%)
	Fear of COVID-19 outbreak	45 (42.06%)
	None	10 (9.35%)
Comorbidities	Present	94 (87.85%)
	Absent	13 (12.15%)
Occupation	Homemaker	24 (22.43%)
	Retired/Unemployed	22 (20.56%)
	Self-employed	49 (45.79%)
	Salaried professional	9 (8.41%)
	Business/Employer	3 (2.80%)
Coping Strategies	Hope for the best	32 (29.91%)
	Remain busy	17 (15.89%)
	Having faith in God/Religion	72 (67.29%)
	Solve issues at my end	74 (69.16%)
	Share feelings with others	59 (55.14%)
	Talk to others	46 (42.99%)
	Avoid thinking about it	18 (16.82%)
	Thinking different things	7 (6.54%)
	Struggling to cope	10 (9.35%)
	Believing that COVID-19 does not exist	5 (4.67%)

Analysing the data, it was found that most people in the sample were aged 60-70 years, had primary school level education, and belonged to the lower socioeconomic status (SES). The majority of the sample population were of Hindu religion residing in a rural setting. Most of the study population lived together with their spouse and were self-employed. The representation from the business owners and salaried professionals was lower. A majority of the population also had additional comorbidities.

We used cut-off scores for conventional severity labels (i.e., normal, mild, moderate, severe, and extremely severe) on the DASS-21 BV to classify anxiety, depression, and stress, as proposed by Lovibond and Lovibond [8]. 57.94% of the participants reported experiencing depressive symptoms. Anxiety symptoms were reported in 39.25% of the population, while 45.79% reported symptoms of stress.

Comparison of depression with sociodemographic variables

A comparison of the depression sub-scale with the various sociodemographic characteristics is given in Table 2. There was a significant increase (p=0.0004) in the prevalence of depression as age increased. 34.5% of the population aged 70+ years had extremely severe depression as compared to only 10.3% of the age group of 60-70 years. Females (72%) also had a significantly (p=0.04) higher prevalence of depression as compared to males (45.61%). Those living separately from their spouses or widowed had a significantly (p= 0.03) higher prevalence of depression. Those with an occupation that caused them to leave their house (32.8%) had a significantly (p=0.03) lower prevalence of depression than those who stayed in the house (63.0%). Sociodemographic factors such as education level, type of family, SES, area of residence, and religion were found to have a non-significant correlation with depression. 

**Table 2:** Comparison of depression with sociodemographic variables *p < .05; **p < .001; ^According to the Revised Modified BG Prasad Socioeconomic Status Classification for October 2023 [11]

Sl. No	Variable		Depression	
			Frequency	Percentage
1	Age (years)**	60-70	34	45.333
		70-80	15	78.947
		80+	6	100
2	Sex*	Male	25	44.643
		Female	30	68.182
3	Education	Never Went to School	16	57.143
		Primary School	26	54.167
		High School	8	42.105
		College	5	100
4	Status of Spouse*	Living Together	35	47.945
		Living Separately/Dead	20	74.074
5	Type of Family	Joint	23	45.098
		Nuclear	32	65.306
6	Socio-Economic Class^	Upper Class	3	75
		Upper Middle Class	3	75
		Middle Class	3	42.857
		Lower Middle Class	17	68
		Lower Class	29	48.333
7	Area Of Residence	Urban	14	60.87
		Rural	41	53.247
8	Religion	Hindu	34	50.746
		Muslim	21	63.636
9	Occupation*	Homemaker/ Unemployed/Retired	27	69.231
		Self-Employed/ Salaried/Employer	28	45.902
10	Comorbidities	Present	52	59.77
		Absent	3	23.077
11	COVID-19 Consequences	Present	50	55.556
		Absent	5	50

Comparison of anxiety with sociodemographic variables

A comparison of the anxiety sub-scale with the various sociodemographic characteristics is given in Table 3. Similar to depression, anxiety levels rose significantly (p=0.002) with age. Of the population in the age group of 70+ years, 69.0% reported anxiety symptoms as compared to only 28.2% of the 60-70-year-olds. Other significant variables were sex (p=0.04), females (52.0%) vs males (28.1%), and surprisingly religion (p=0.0002); those practising Islam (65.0%) vs Hinduism (23.9%). Educational status, status of spouse, type of family, SES, area of residence, and employment status were found to have a non-significant correlation with anxiety.

**Table 3:** Comparison of anxiety with sociodemographic variables *p < .05; **p < .001; ^According to the Revised Modified BG Prasad Socioeconomic Status Classification for October 2023 [11]

Sl. No	Variable		Anxiety	
			Frequency	Percentage
1	Age (years)**	60-70	19	25.333
		70-80	10	52.632
		80+	6	100
2	Sex*	Male	15	26.786
		Female	20	45.455
3	Education	Never Went to School	12	42.857
		Primary School	14	29.167
		High School	7	36.842
		College	2	40
4	Status of Spouse	Living together	24	32.877
		Living separately/ Dead	11	40.741
5	Type of Family	Joint	13	25.49
		Nuclear	22	44.898
6	Socio-Economic Class^	Upper Class	1	25
		Upper Middle Class	1	25
		Middle Class	3	42.857
		Lower Middle Class	11	44
		Lower Class	19	31.667
7	Area Of Residence	Urban	10	43.478
		Rural	25	32.468
8	Religion**	Hindu	16	23.881
		Muslim	19	57.576
9	Occupation	Homemaker/ Unemployed/Retired	21	53.846
		Self-Employed/ Salaried/Employer	14	22.951
10	Comorbidities	Present	35	40.23
		Absent	-	-
11	COVID-19 Consequences	Present	33	36.667
		Absent	2	20

Comparison of stress with sociodemographic variables

A comparison of the stress sub-scale with the various sociodemographic characteristics is given in Table 4. As expected, stress levels had a linear relation with age (p=0.00009). Of the 60-70-year-old population, 39.7% had stress as compared to 62.1% of the population aged 70+ years. Interestingly, the severest form of stress (ie, extremely severe categorization) was more common in the younger age group. Sociodemographic variables that had a significant correlation were sex (p=0.03), status of spouse (p= 0.01), place of residence (p=0.02), and religion (p=0.01). Respective proportions of stress symptoms were female (58.0%) vs male (35.1%), those living separately from their partners (67.6%) vs living with their partners (35.6%), those residing in urban environments (65.5%) vs those living in rural settings (38.5%), and those practising Islam (67.5%) vs Hinduism (32.8%).

**Table 4:** Comparison of stress with sociodemographic variables *p < .05; **p < .001; ^According to the Revised Modified BG Prasad Socioeconomic Status Classification for October 2023 [11]

Sl. No	Variable		Stress	
			Frequency	Percentage
1	Age (years)**	60-70	30	40
		70-80	7	36.842
		80+	6	100
2	Sex*	Male	19	33.929
		Female	24	54.545
3	Education	Never Went to School	16	57.143
		Primary School	19	39.583
		High School	6	31.579
		College	2	40
4	Status of Spouse*	Living Together	26	35.616
		Living Separately/Dead	17	62.963
5	Type of Family	Joint	18	35.294
		Nuclear	25	51.02
6	Socio-Economic Class^	Upper Class	2	50
		Upper Middle Class	2	50
		Middle Class	3	42.857
		Lower Middle Class	11	44
		Lower Class	25	41.667
7	Area Of Residence*	Urban	13	56.522
		Rural	30	38.961
8	Religion*	Hindu	22	32.836
		Muslim	21	63.636
9	Occupation	Homemaker/ Unemployed/Retired	23	58.974
		Self-Employed/ Salaried/Employer	20	32.787
10	Comorbidities	Present	42	48.276
		Absent	1	7.692
11	COVID-19 Consequences	Present	38	42.222
		Absent	5	50

COVID-19 consequences

Twelve (11.2%) participants had experienced COVID-19 symptomatology and 10 (9.3%) had been hospitalised. A majority of the sample population (n=86, 80.3%) had experienced a loss of income due in part to their being unable to participate in daily economic activities as a result of social shunning (n=21, 19.6%), and fear of a COVID-19 outbreak in their communities (n=45, 42.1%). Thirteen (12.1%) participants had lost a loved one during the pandemic due to COVID-19.

Coping strategies

The most frequently used coping strategy was to solve problems they faced daily (n=74, 69.2%), closely followed by praying and participating in religious activities (n=72, 67.3%). Other popular strategies were sharing feelings with friends and family, and talking to other people. Out of 107 total participants, 91 (85.1%) employed more than one coping strategy to try and tackle the unprecedented levels of stress, anxiety, and depression due to the pandemic.

## Discussion

The present research aimed to investigate the prevalence of mental health problems, potential risk factors, and coping mechanisms employed by individuals amidst the COVID-19 pandemic, and examine their sociodemographic correlation. This study specifically focused on a high-risk population, elderly patients aged 60 years and above who were already receiving medical care for other health conditions.

The findings of this study revealed that 43.9%, 32.7%, and 34.6% of the sampled group were moderately to extremely severely depressed, anxious, and stressed, respectively. Comparable observations have been documented in a limited number of prior studies [12,13], albeit with considerably lower reported rates of depression. Jain et al. [12] conducted a community-based cross-sectional study conducted in Mumbai, India, in which they used the Geriatric Depression Scale (GDS) [14] to assess depression, among the geriatric population residing in urban slums and found that 45.9% of individuals were suffering from depression. Dasgupta et al. [13] used the shorter format of the GDS (GDS-15) [15] and found a prevalence of 46.9% for elderly people residing in the slums of Kolkata, India. A systematic review reported a rate of 31.74% for the prevalence of depression among the old age population and a rate of 40.78% for those from developing countries like India [16]. Pilania et al. estimated the prevalence of depression among the Indian elderly population at 34.4% [2]. This is still much lower than our finding (57.94%) possibly due to the COVID-19 pandemic. Disasters which involve such loss of lives, and widespread disruptions are usually short-term crisis events, but during the pandemic, people experienced an ongoing and pervasive sense of loss for long periods of time with tragic deaths and threatened loss of loved ones. People were continuously fearful of getting infected, and hence experienced and partook in social shunning. The loss of a sense of normalcy due to the loss of jobs and financial security, and the loss of physical contact with family members and social networks led to an increase in mental illness during this period.

A similar trend can be seen for anxiety disorders, wherein authors reported a 17.67% prevalence for anxiety disorders among the geriatric population [16]. Our study in comparison concluded a prevalence of 39.25%. Regional variations in the prevalence of mental health concerns among the elderly population may be attributed to cultural disparities in the expression of distress, the availability of support systems, variations in research methodologies, and other potential confounding variables specific to the sampled population. Therefore, conducting further research in this area could provide valuable insights into the underlying factors influencing these variations. Common risk factors for higher levels of depression and anxiety included being female, living in a rural environment, being unmarried, and living in a nuclear family [2,13].

This study highlights a significant prevalence of mental health issues among the elderly population, underscoring the necessity for comprehensive investigations and the implementation of well-structured approaches to effectively address these concerns. These findings play a crucial role in aiding countries in policy development and strengthening their welfare programmes [17]. Failing to take appropriate and timely action may give rise to additional complications, such as increased substance abuse and a heightened risk of suicides, as observed in the aftermath of disasters [18]. Consequently, it is crucial to prioritise proactive interventions and establish preventive measures to mitigate the potential consequences associated with untreated mental health problems among the elderly population.

Risk factors

The findings of this study revealed significant associations between mental health issues and various demographic factors among the elderly population. Increasing age and female gender were associated with higher levels of depression, anxiety, and stress. This finding was in line with the National Mental Survey of India, 2016, which reported that females are more likely to be suffering from depressive disorders and anxiety than males [19]. Specifically, individuals aged 70 and above were far more likely to be suffering from severe and extremely severe depression, anxiety, and stress. Interestingly those practicing Islam had a significantly higher prevalence of anxiety and stress. A similar finding was reported by Gupta et al., who found that the disparity in self-reported mental health remained even after controlling for education and SES [20]. Those who were single or living separately from their spouses, or who had experienced spousal bereavement were found to be more likely to exhibit symptoms of depression and stress. This could be attributed to the inability to exercise some of the more common coping strategies such as sharing feelings with family and talking to others. Similarly, individuals who were unemployed, retired, or homemakers and did not engage in work-related activities outside their homes demonstrated an increased vulnerability to depression. A possible explanation could be that leaving the house for work forces one to be engaged in conversation with others throughout the week.

These specific groups appeared to be particularly affected by the lack of coping mechanisms, such as the ability to engage in conversations and express emotions, exacerbating their pre-existing mental health disorders in addition to the general concerns associated with the COVID-19 pandemic. The absence of social contact likely contributed to the deterioration of their mental well-being.

Coping

The presence of effective coping strategies in stressful situations is crucial, as they can serve as preventive measures against stress-related psychiatric disorders. While individual susceptibility to stress and specific circumstances play a role, the utilization of coping strategies is anticipated to provide assistance. It is known that people use various coping methods in crisis or disaster situations [21], as also observed in this study. The results suggested that solving problems they faced daily was the most frequent way of coping, followed by praying and participating in religious activities, as mentioned in Table 1. Additionally, our study indicated that nearly everyone employed some form of coping mechanism, to address the unparalleled levels of stress, anxiety, and depression caused by the pandemic. 

Therefore, in line with the findings of the study, it would be beneficial to offer information on coping mechanisms and effective strategies to manage mental health disorders. Resources should be first assigned to the at-risk populations to decrease individual and social morbidity. As secondary stressors arise, such as economic hardships, job losses, and bereavement, the burden of concerns may intensify. Thus, it is essential to educate individuals about the resources at their disposal and provide practical approaches to address and cope with these emerging issues.

Intervention

The impact of humanitarian crises and disasters on the mental well-being of the population is widely recognised, necessitating the development and implementation of comprehensive and timely interventions, both in the short and long term. It is evident that alongside the COVID-19 pandemic, a significant mental health crisis has unfolded, which is expected to persist worldwide for an extended period.

In the acute phase of any pandemic, when stress and anxiety levels in society are heightened, it becomes crucial for any emergency response to incorporate the management of the mental health crisis [22]. This holds true not just for individuals exhibiting COVID-19 symptoms, but also for their families. When patients with COVID-19 are hospitalised, it is highly unlikely that their family members will be permitted to visit them, thereby exacerbating the stress and anxiety experienced by both the patients and their loved ones.

The elderly also already face a huge burden of diseases, which is exacerbated by psychological problems. Research shows that depressive and anxiety disorders significantly worsen the prognosis of several other diseases [23]. Given the widespread prevalence of mental health concerns affecting the geriatric population, interventions should prioritize addressing their needs. This becomes even more critical considering that existing mental health services may become overwhelmed by the demands of diagnosing and treating these mental health issues.

In the context of this study, it is essential to explore the effectiveness and applicability of specific therapeutic approaches, such as mindfulness, cognitive-behavioural therapy, and bibliotherapy, in addressing mental health challenges within the current circumstances. These evidence-based therapies have shown promise in various mental health contexts and warrant investigation for their utility in the specific context of the COVID-19 pandemic.

Furthermore, considering the diverse population in India, it is crucial to ensure that the available support services are accessible in different languages. While written and video materials are already available [24], it is necessary to conduct assessments to determine their utilization, effectiveness, and, most importantly, authenticity. Ensuring the reliability and cultural relevance of these resources will enhance their utility in supporting the mental health and well-being of the population.

The internet has long served as a medium for providing assistance, and its potential can be harnessed to deliver accurate information, dispel myths, and disseminate up-to-date data pertaining to the pandemic. Online platforms offer opportunities for the provision of essential services, including counseling, resilience training, and psychotherapy.

Limitations

This study acknowledges several limitations despite the rigorous efforts of the authors. The composition of the sample population has some bias in that it was notably deficient of individuals who possessed a college education, resided in urban environments, belonged to the middle class or higher SES, or held occupations as employers or salaried professionals. Consequently, the sample may not be representative of the broader elderly patient population, as sampling was purposeful rather than random. The findings would have benefited from a larger sample size, which could have enhanced the accuracy of the results and facilitated exploration of potential regional and cultural variations in symptom presentation, thus warranting consideration in future research endeavours.

As the study was conducted in a hospital setting, the results cannot be generalised to the general population. To validate the findings of our study, further similar studies can be undertaken in other hospitals and also in the community setting.

Additionally, being reliant on self-report questionnaires, the study was susceptible to response biases. Participants may have provided responses that align with their comfort levels, despite assurances regarding the anonymity of data. This potential bias should be taken into account when interpreting the findings.

## Conclusions

The impact of the COVID-19 pandemic on the mental health of the elderly population within the community has been substantial, with a notable increase in prevalence of symptoms related to anxiety, depression, and stress. Consequently, it is imperative to establish comprehensive strategies aimed at effectively managing the scale of mental health morbidities experienced by this demographic.

A crucial aspect of addressing this issue is the implementation of public education initiatives that provide individuals with knowledge about effective coping strategies. Equipping the elderly population with the necessary tools to navigate the challenges posed by the pandemic can empower them to better manage their mental well-being. By adopting a proactive and sustained approach, the impact on mental health can be minimized, fostering better overall well-being among the elderly population.

## Figures and Tables

**Figure 1:**
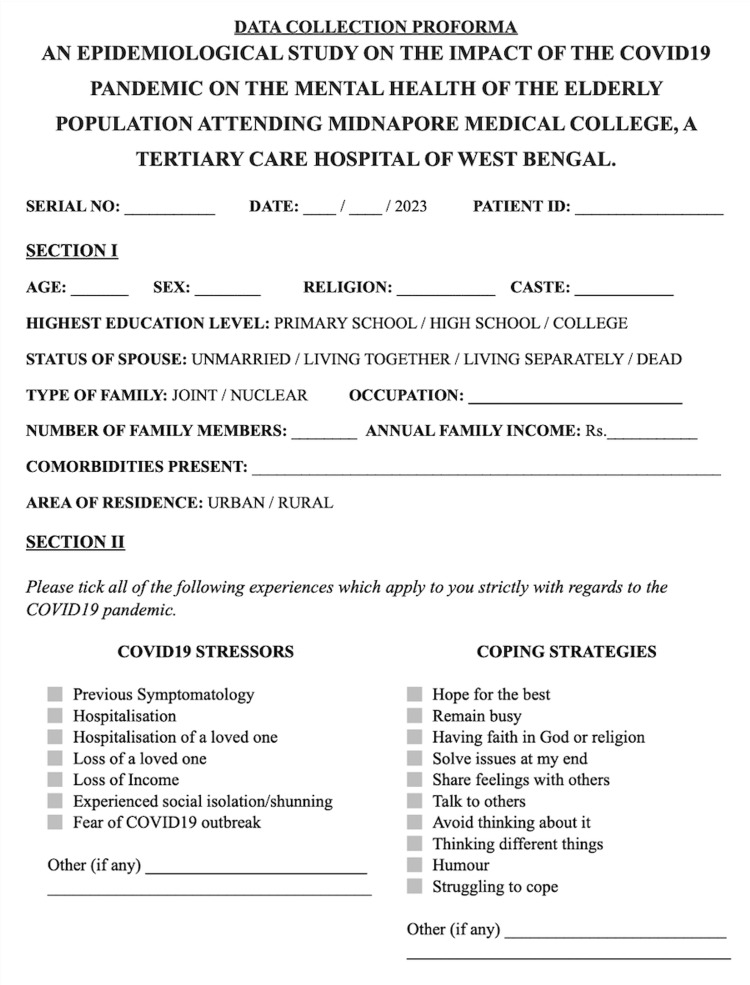
Data Collection Proforma Section I and II

**Figure 2:**
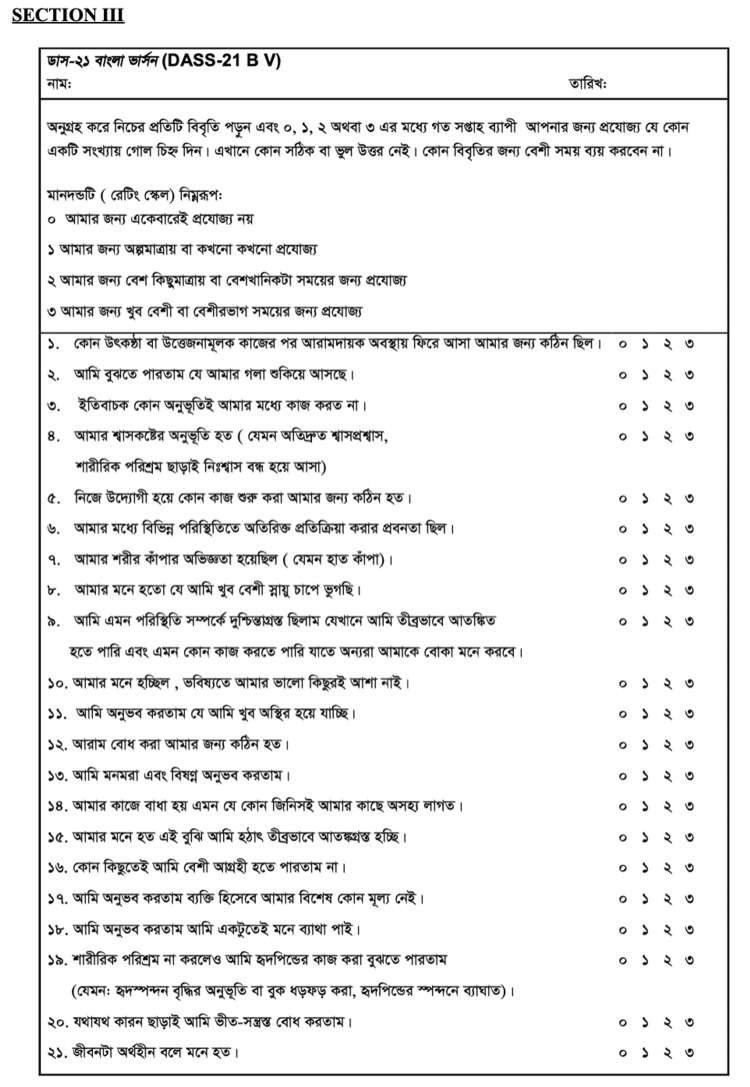
Data Collection Proforma Section III (in Bangla)

## Appendices

Data collection proforma (sections I and II)

Data collection proforma (section III)
